# Prevalence and risk factors of home delivery among rural women: evidence from 28 African countries

**DOI:** 10.1186/s12978-026-02386-x

**Published:** 2026-06-20

**Authors:** Ebenezer Kwesi Armah-Ansah, Deborah Okeke-Obayemi, Sylvester Okeke, Eugene Budu, Charity Oga-Omenka, Edward Kwabena Ameyaw

**Affiliations:** 1 School of Public Health Sciences, University of Waterloo, Waterloo, Canada; 2 School of Population Health, UNSW Sydney, Sydney, Australia; 3 Centre for Social Research in Health, UNSW Sydney, Sydney, Australia; 4 School of Social Sciences, UNSW Sydney, Sydney, Australia; 5 School of Education, Macquarie University, Sydney, Australia; 6 Institute of Human Virology, Abuja, Nigeria; 7 Institute of Policy Studies and School of Graduate Studies, Lingnan University, Tuen Mun, Hong Kong; 8 L & E Research Consult Ltd, Wa, Upper West Region Ghana

**Keywords:** DHS, Childbirth, Preference, Pregnancy intention, Reproductive health

## Abstract

**Background:**

Home delivery continues to be a significant factor contributing to maternal mortality in Africa, particularly among rural women with limited access to skilled birth attendants and healthcare facilities. Factors influencing home delivery operate at individual, household, and community levels. Therefore, this study aimed to examine the prevalence and risk factors of home delivery among rural women in 28 African countries.

**Methods:**

This retrospective cross-sectional study analyzed the most recent Demographic and Health Surveys (2011–2024) from 28 African countries. The weighted sample included 103,011 rural women of reproductive age. We performed descriptive analysis, chi-square tests, and binary logistic regression. Results are presented as frequencies, percentages, and odds ratios (ORs) with 95% confidence intervals (CIs). Statistical significance was set at *p* < 0.05.

**Results:**

The overall prevalence of home delivery among rural women in Africa was 34.01% [95% CI: 23.33%-35.26%], ranging from 5.91% in Rwanda to 85.19% in Chad. Women with mistimed pregnancy [aOR = 0.79, 95% CI = 0.76–0.82] and those with unwanted pregnancy [aOR = 0.86, 95% CI = 0.81–0.92] had lower odds of home delivery. Risk factors included having four or more births [aOR = 2.04, 95% CI = 1.91–2.17], no/other religion [aOR = 2.41, 95% CI = 2.24–2.56] and those in Central Africa [aOR = 2.09, 95% CI = 1.98–2.19].

**Conclusion:**

This research reveals that home delivery remains prevalent among rural women in Africa, with significant between-country disparities. Key risk factors include high parity, no/other religion, and Central African residence. Programs should prioritize multiparous women and expand maternal health services across all religious groups. Additionally, context-specific policies and targeted investments are needed in Central Africa to address regional disparities.

## Introduction

Despite substantial gains in skilled birth attendance (SBA) across Africa from approximately 52% to 73% over the past two decades, home delivery remains a persistent and deeply entrenched practice among rural women in the region [1–6]. Progress has been constrained by enduring structural, health system, and social barriers across many African countries [3, 4, 7, 8]. Although maternal mortality is a global concern, its burden is disproportionately concentrated in Africa, where over 300,000 women die annually from pregnancy and childbirth complications [3, 7, 9].This persistently high mortality burden underscores the urgent need for country-specific, effective, and multisectoral strategies to reduce high maternal mortality ratio (MMR) in this setting [3, 4, 7].

Empirical evidence indicates complex interplay of structural, health system, and social determinants that shape place of delivery [4–12]. Specifically, inadequate access to quality healthcare services, limited availability of skilled birth attendants, and social determinants of health such as poverty, gender inequality and low maternal education have all been implicated in high home delivery rates [5, 10]. These challenges are particularly pronounced in settings where there is limited SBA and healthcare facilities [4, 10–12]. A large body of evidence shows persistent disparities between urban and rural areas in maternal health outcomes [11–14]. These studies show that rural women face disproportionately higher odds of home delivery due to contextual factors such as low socioeconomic status, problems with distance to health facilities, transportation barriers, and limited SBA availability [15–17].

Beyond structural and geographic barriers, a woman’s pregnancy intention has emerged as an important determinant of place of delivery [18, 19]. For instance, unintended pregnancies have been linked to higher home delivery rates in Uganda [18] and Tanzania [19], lower antenatal care (ANC) utilization, stillbirths, and neonatal death [20]. Additionally, women with unintended pregnancies may delay care or face barriers to health facility delivery due to increased maternal depression and pregnancy complications [21–23]. Empirical evidence indicates that maternal and paternal education, limited ANC visits, and community poverty increase likelihood for home delivery [24, 25].

Despite this body of evidence, existing studies on home delivery in Africa are largely limited to single-country analyses or subnational samples [17–19]. This limitation underscores the need for up-to-date, context-specific empirical research to inform policies and programs capable of meeting established maternal health targets [4, 7].

A comprehensive understanding of factors influencing home delivery among women of reproductive age in rural areas is important for informing evidence-based policies and programs aimed at improving maternal health outcomes in Africa. Therefore, this study aims to estimate the prevalence and identify risk factors of home delivery among rural women of reproductive age across 28 African countries using recent Demography and Health Survey (DHS) data.

## Materials and methods

### Data source and study population

This retrospective cross-sectional study used secondary data extracted from the women’s recode file (IR) of the most recent DHS conducted between 2011 and 2024 in 28 World Health Organization (WHO) African countries. These 28 African countries were included in the study because they had all the variables of interest for this study. Although the DHS program conducts surveys approximately every five years, surveys are conducted independently by country. Therefore, we used the single most recent survey per country, resulting in varying survey years (ranging from 2011 to 12 in Congo to 2024 in Angola, Lesotho, Mali, Nigeria, and Zambia), reflecting differences in the timing of each country’s most recent data collection rather than repeated measures from the same country over time. To ensure cross-country comparability, all data were harmonized using uniform variable definitions, consistent recoding, and pooled analysis with applied sampling weights.

The DHS is a nationally representative survey undertaken in more than 90 low- and middle-income countries (LMICs) in Asia and Africa [26, 27]. The DHS has been in existence since 1984, is conducted every five years, and focuses on health-related variables including mortality, morbidity, use of family planning services, fertility, nutrition, and maternal and child health [24]. The DHS uses a descriptive cross-sectional design and a two-stage stratified selection procedure to recruit respondents (i.e., enumeration areas [EAs]) [26, 28]. While DHS surveys are representative at the national level and weighted, this analysis was restricted to rural African women in their reproductive age (15–49 years) with at least a birth history in the 5 years prior to the collection of the data.

**Fig. 1:**
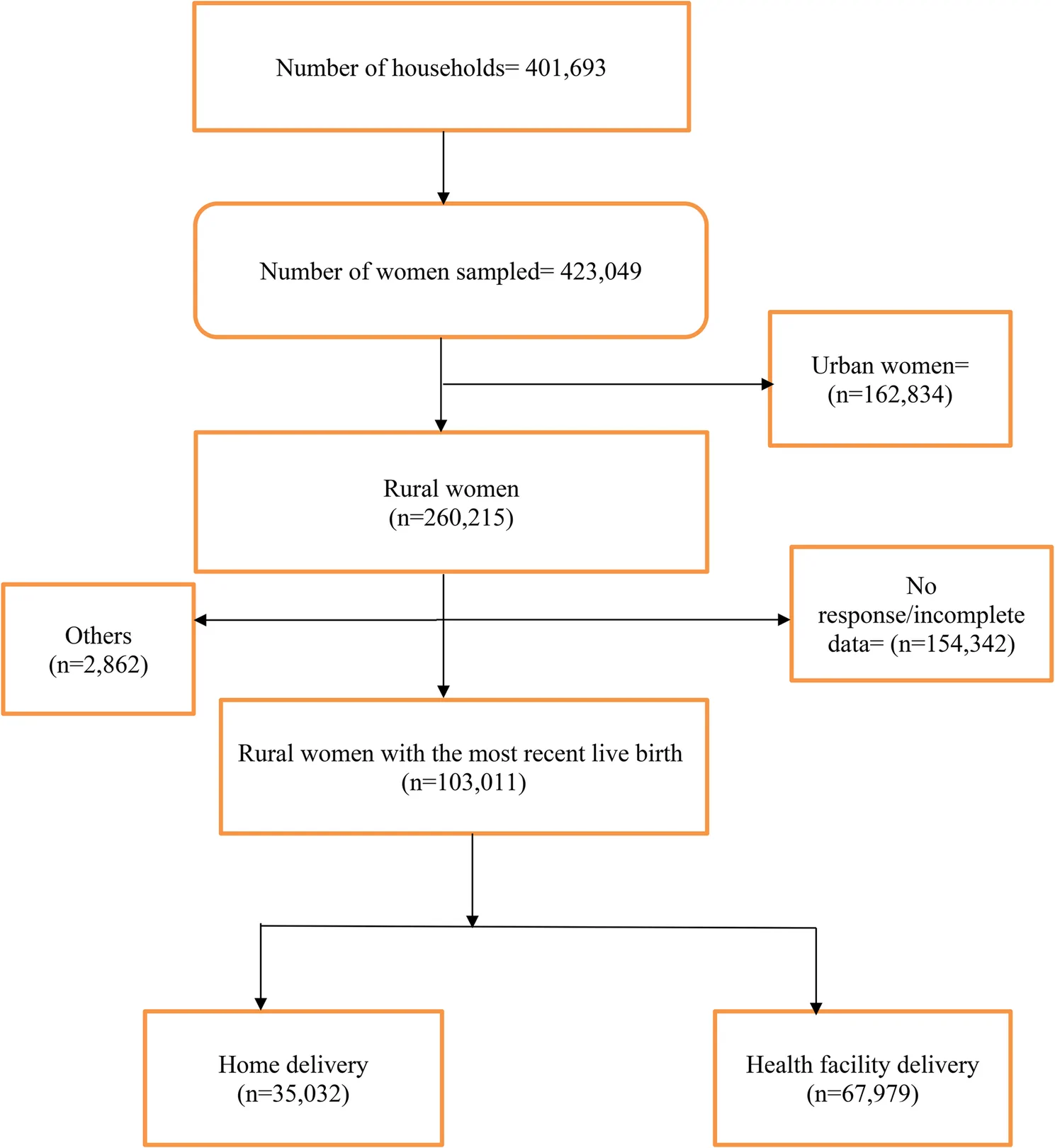
A framework showing the sampling procedure for selecting the study participants

The individual sampling weights from DHS were applied to this narrowed-down analytical sample. Women reporting ‘other’ place of delivery (*n* = 2,862) and those with no response or incomplete data (*n* = 154,342) were excluded from the analysis (complete-case analysis), resulting in a final weighted analytic sample of 103,011 rural women with a recent live birth. The datasets supporting this study are freely available from the DHS Program at https://dhsprogram.com/data/available-datasets.cfm. We relied on the Strengthening the Reporting of Observational Studies in Epidemiology (STROBE) statement guidelines in drafting this manuscript [29]. A detailed description of the sample extracted for the study can be found in Table 1, which summarizes the survey years, weighted sample sizes, and percentages for each of the 28 African countries included. The sampling procedure used to select the study participants is illustrated in Fig. 1, showing the stepwise derivation of the analytical sample.

**Table 1:** Detailed description of the weighted study sample

	Survey country	Year of survey	Weighted sample (*N*)	Weighted percentage (%)
1.	Angola	2023–2024	1,519	1.47
2.	Benin	2017-18	4,645	4.51
3.	Burkina Faso	2021	4,220	4.10
4.	Burundi	2016–2017	6,030	5.85
5.	Cameroon	2018	2,696	2.62
6.	Chad	2014–2015	2,534	3.16
7.	Comoros	2012	928	0.90
8.	Congo	2011-12	3,330	3.20
9.	Congo DR	2013-14	6,465	6.28
10.	Cote d’Ivoire	2021	2,825	2.74
11.	Ethiopia	2016	5,195	5.04
12.	Gabon	2012	928	0.90
13.	Ghana	2022	2,597	2.52
14.	Guinea	2018	3,565	3.46
15.	Lesotho	2023–2024	730	0.71
16.	Liberia	2019-20	1,812	1.76
17.	Madagascar	2021	5,509	5.35
18.	Malawi	2015–2016	8,900	8.64
19.	Mali	2023–2024	5,490	5.33
20.	Mozambique	2022–2023	2,390	2.32
21.	Namibia	2013	765	0.74
22.	Nigeria	2024	7,906	7.67
23.	Rwanda	2019-20	3,849	3.74
24.	Sierra Leone	2019	3,793	3.68
25.	Togo	2013-14	3,179	3.09
26.	Uganda	2016	6,539	6.35
27.	Zambia	2024	2,351	2.28
28.	Zimbabwe	2015	2,351	2.28
	African countries	2011–2024	103,011	100.0

### Description of variables

#### Dependent variable

The outcome variable “home delivery” was derived from the “place of delivery” question. For this study, women who delivered at home (where the birth occurred at the respondent’s home and other home) were put in the category “yes” and were given the code ‘1,’ whereas women who had deliveries in health facilities (hospital, health centre, or health post) were put in the category “no,” and this was coded as ‘0.’

Women who indicated ‘others’ as a response category were excluded from this study as this category does not align with either home or facility deliveries and therefore does not contribute to the study’s objective.

#### Independent variables

Pregnancy intention was treated as the main explanatory variable. The remaining variables were considered risk factors associated with home delivery. The pregnancy intention variable was derived from the question of whether the respondent “wanted the last child.” Women were asked whether the pregnancy had been planned (desired at that time), mistimed (the pregnancy occurred earlier than desired), or unwanted (the pregnancy occurred when no children, or no more children, were desired). These response categories were recoded as “1 = planned,” “2 = mistimed,” and “3 = unwanted.” This categorization was informed by literature [30–33].

### Risk factors

Based on the literature [9, 34–37], a total of seventeen risk factor variables were included. These risk factors were grouped into individual-level and contextual-level variables. The individual-level variables are personal characteristics or experiences that influence the relationship between an independent variable and a dependent variable, while the contextual-level variables are factors in setting, environment, or circumstances that influence the relationship between an independent variable and a dependent variable [38]. Thirteen of the seventeen risk factor variables were therefore considered as individual-level factors, and the remaining four were contextual-level variables (see Table 2). We maintained the existing DHS coding of these individual-level variables: age, sex of household head, health insurance coverage, and wealth index. Maternal education and their partner’s education were recoded as “no education,” “primary education,” and “secondary/higher.” Marital status was recoded as “married” and “cohabitation,” while employment status was recoded as “not working” and “working.” Religion was recorded as “Christian,” “Islam,” “Traditional,” and “No/Other.” Antenatal care visits were recoded as “less than four” and “four or more,” whereas healthcare decision-making capacity was recoded as “alone” and “not alone.”

**Table 2:** Cross-tabulation results between explanatory variables and home delivery among rural women in Africa (Weighted, *N* = 103,011)

Variables	Weighted *N*	Weighted (%)	Home delivery (%)		χ2, *p*-value
			No (%)	Yes (%)	
Pregnancy Intention					**707.98,** ***p* < 0.001**
Planned	77,694	75.4	64.2	35.8	
Mistimed	18,513	18.0	72.6	27.4	
Unwanted	6,804	6.6	68.7	31.3	
Age					**116.19**, ***p* < 0.001**
15–19	7,090	6.8	65.1	34.9	
20–24	23,352	22.7	67.8	32.2	
25–29	25,767	25.0	66.1	33.9	
30–34	20,957	20.3	66.7	33.3	
35–39	15,501	15.0	65.7	34.4	
40–44	7,773	7.6	61.9	38.1	
45–49	2,571	2.5	59.3	40.7	
Education					**20.94**, ***p* < 0.001**
No education	45,284	37.3	53.7	46.4	
Primary	38,067	31.7	72.4	27.6	
Secondary+	19,660	31.0	81.9	18.1	
Partner’s Education					**16.05**, ***p* < 0.001**
No education	39,290	38.1	54.6	45.4	
Primary	34,641	33.6	69.4	30.6	
Secondary+	29,080	28.3	77.4	22.6	
Marital Status					**471.15**, ***p* < 0.001**
Married	81,852	79.5	64.4	35.6	
Cohabitation	21,159	20.5	72.1	27.9	
Employment status					**962.68**, ***p* < 0.001**
Not working	28,852	28.0	58.3	41.7	
Working	74,081	72.0	69.0	31.0	
Parity					**6.26**, ***p* < 0.001**
One birth	17,246	16.7	74.7	25.3	
Two births	18,460	17.9	70.1	29.9	
Three births	16,824	16.4	67.1	32.9	
Four or more births	50,481	45.0	61.1	38.9	
Mass Media					**13.06**, ***p* < 0.001**
No	51,538	50.1	57.0	43.0	
Yes	51,473	49.9	75.0	25.0	
ANC visits					**29.91**, ***p* < 0.001**
Less than 4	49,378	47.9	51.6	48.4	
4 or more	53,633	52.1	79.2	20.8	
Health insurance coverage					**8.44**, ***p* < 0.001**
No	94,227	91.5	63.9	36.1	
Yes	8,784	8.5	88.4	11.6	
Healthcare decision-making capacity					**151.41**, ***p* < 0.001**
Alone	16,098	15.6	70.1	29.9	
Not Alone	86,913	84.4	65.2	34.8	
Religion					**17.95**, ***p* < 0.001**
Christians	62,032	60.2	74.0	26.0	
Islam	34,338	33.3	54.8	45.2	
Traditional	2,394	2.3	57.1	42.9	
No/other religion	4,247	4.2	45.1	54.9	
Wealth index					**14.14**, ***p* < 0.001**
Poorest	30,921	30.0	55.7	44.3	
Poorer	28,853	28.0	63.7	36.3	
Middle	23,172	22.5	70.6	29.5	
Richer	14,732	14.3	77.2	22.8	
Richest	5,333	5.2	88.1	11.9	
Sex of household head					**244.60**, ***p* < 0.001**
Male	89,902	87.3	64.9	35.1	
Female	13,109	12.7	73.9	26.1	
Community-level variables					
Accessing healthcare					**6.26**, ***p* < 0.001**
With big problem	11,709	11.4	51.2	48.8	
Without a big problem	91,302	88.6	67.9	32.1	
Community literacy level					**16.32**, ***p* < 0.001**
Low	38,723	37.5	54.5	45.5	
Medium	32,703	31.8	69.3	30.7	
High	31,585	30.7	76.7	23.3	
Community socioeconomic status					**13.06**, ***p* < 0.001**
Low	71,230	69.2	60.2	39.8	
Moderate	8,367	8.1	82.8	17.2	
High	23,414	22.7	77.3	22.7	
Sub-region					**7.08**, ***p* < 0.001**
West Africa	39,259	38.1	64.2	35.8	
Central Africa	14,988	14.6	55.5	44.5	
East Africa	44,754	43.4	70.0	30.0	
Southern Africa	4,010	3.9	78.2	21.8	

*p*-value less than 0.05 indicates statistical significance

Mass media was created as a composite variable from three variables measuring women’s mass media exposure: (1) frequency of listening to radio, (2) frequency of watching television, and (3) frequency of reading newspapers and magazines. These three variables had the same response options: “not at all,” “less than once a week,” and “at least once a week.” Based on the literature [39, 40], we grouped the response options into “no,” which meant no mass media exposure (not at all), and “yes,” which meant mass media exposure (less than once a week and at least once a week).

The contextual variables included access to healthcare, place of residence, community literacy level, and community socioeconomic status. We maintained the existing coding for place of residence found in the DHS datasets. Access to healthcare was generated from four variables (getting the money needed for treatment, distance to the health facility, getting permission to go, and not wanting to go alone). These questions had the same response options: “as a big problem” and “not a big problem.” Based on the literature, we coded these questions as “accessing healthcare” with the response options as “0” as a big problem and “1” as not a big problem [41]. Women who reported at least one of these as a ‘big problem’ were classified as having poor access to healthcare, whereas those who reported ‘not a big problem’ were classified as having good access to healthcare [42].

Community literacy level was constructed by aggregating individual women’s literacy status within each DHS cluster. Community literacy level was calculated as a proportion of women who could either read and write or could not read and write at all. The proportion of women who could read and write was computed for each cluster and categorized into low, medium, and high literacy levels using tertile cut-off points, such that each category contained approximately one-third of the clusters.

Community socioeconomic status was assessed based on the employment, wealth, and education of the women who resided in that community. We utilized principal component analysis to assess the number of women who were unemployed, illiterate, and poor. A standardized score was established with a mean score of 0 and a standard deviation. The scores were then split into three tertiles: 1 (most disadvantaged), 2 (middle disadvantaged), and 3 (least disadvantaged), with tertile “1” representing a low socioeconomic status, tertile “2” representing moderate socioeconomic status, and tertile “3” representing high socioeconomic status [43]. The studied countries were grouped into their respective geographical regions: West Africa, Central Africa, East Africa, and Southern Africa.

### Statistical analysis

**Fig. 2:**
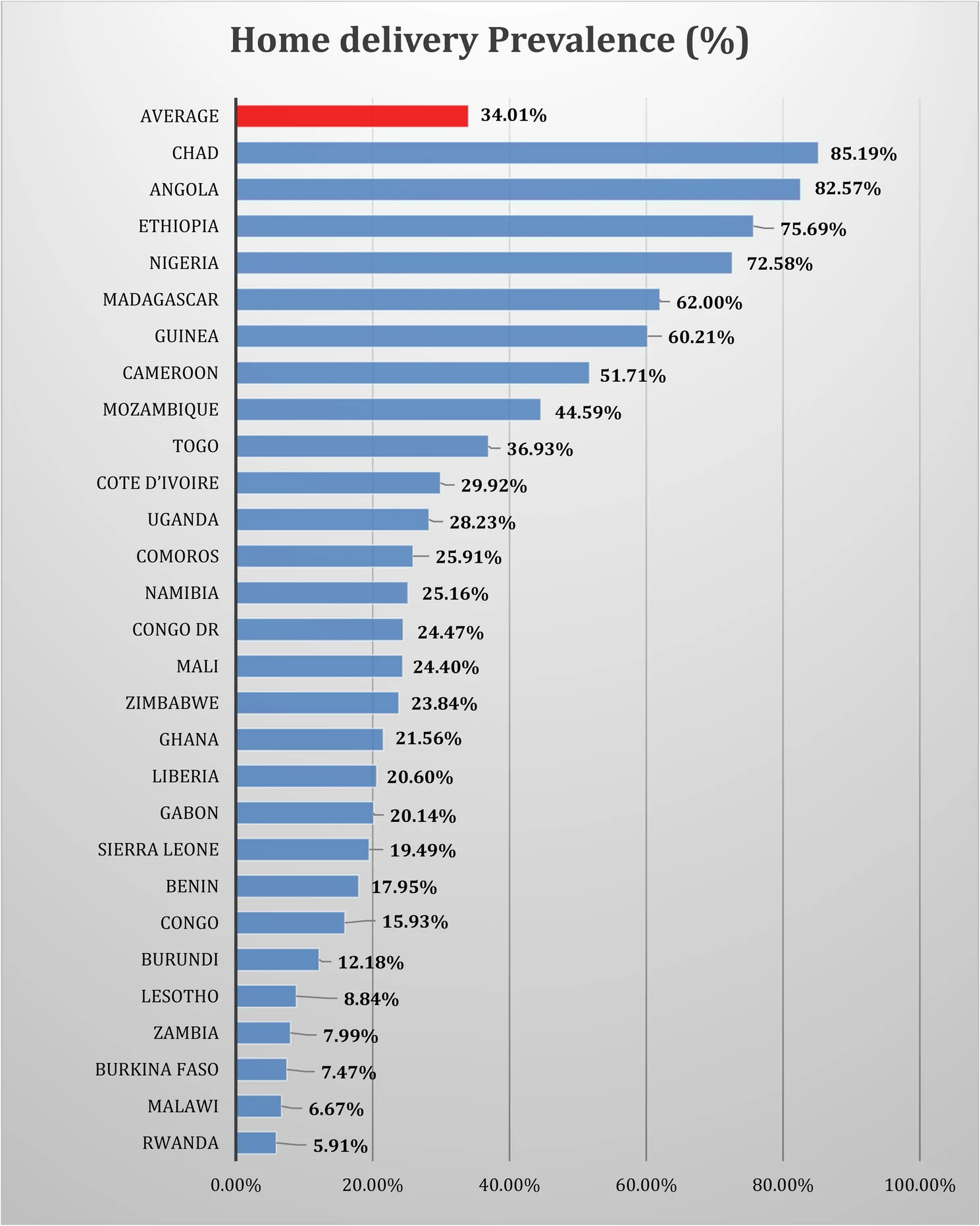
Prevalence (%) of home delivery among women in rural areas of Africa

Data analyses for this study were performed using Stata version 14.2 for MacOS. Three levels of analyses (descriptive, bivariate, and binary logistic regression) were conducted. The analysis began with appending the datasets, and this generated a total weighted sample (see Table 1). At the univariate level, a graphical representation of the prevalence of home delivery among women in rural areas of Africa was displayed using a bar chart (see Fig. 2). Table 2 displays the weighted frequencies and percentages of the main explanatory variable and risk factor variables. Cross-tabulation with Pearson’s chi-square (X²) tests was performed, and associations were considered statistically significant at *p* < 0.05.

Statistically significant variables in the bivariate analysis were moved to the binary logistic regression models (Table 3). A three-model binary logistic regression was used to examine associations between the explanatory variables and home delivery. The first model, Model I, had the key explanatory variable only against the outcome variable. In the second model, Model II, the individual-level risk factor variables were added to the model, while in Model III, which is considered the complete model, the contextual variables were included. The statistical significance level was set at *p* < 0.05, and the results were presented as crude odds ratios (cORs) and adjusted odds ratios (aORs) with 95% confidence intervals (CIs). Associations were considered statistically significant if the 95% confidence interval did not include the null value of 1.00.

**Table 3:** Association between pregnancy intention and home delivery among women in rural Africa adjusting for risk factors

Variables		Model I cOR (95% CI)	Model II aOR (95% CI)	Model III aOR (95% CI)
Pregnancy Intention				
Planned		Ref	Ref	Ref
Mistimed		0.63*** (0.61–0.65)	0.76*** (0.73–0.79)	0.79*** (0.76–0.82)
Unwanted		0.76*** (0.72–0.81)	0.81*** (0.81–0.86)	0.86*** (0.81–0.92)
*Individual/household level variables*				
Age				
15–19			Ref	Ref
20–24			0.79*** (0.74–0.84)	0.84*** (0.78–0.90)
25–29			0.67*** (0.62–0.72)	0.72*** (0.68–0.78)
30–34			0.57*** (0.52–0.61)	0.63*** (0.58–0.69)
35–39			0.55*** (0.50–0.60)	0.61*** (0.56–0.67)
40–44			0.59*** (0.54–0.64)	0.65*** (0.59–0.71)
45–49			0.61*** (0.54–0.68)	0.68*** (0.60–0.76)
Education				
No education			Ref	Ref
Primary			0.72*** (0.69–0.75)	0.71*** (0.68–0.74)
Secondary+			0.65*** (0.61–0.68)	0.68*** (0.64–0.72)
Partner’s Education				
No education			Ref	Ref
Primary			0.93*** (0.89–0.96)	0.88*** (0.84–0.91)
Secondary+			0.88*** (0.85–0.92)	0.80*** (0.76–0.84)
Marital Status				
Married			Ref	Ref
Cohabitation			0.90*** (0.86–0.93)	0.82*** (0.79–0.86)
Employment				
Not working			Ref	Ref
Working			0.78*** (0.75–0.80)	0.80*** (0.78–0.83)
Parity				
One birth			Ref	Ref
Two births			1.42*** (1.35–1.51)	1.39*** (1.31–1.47)
Three births			1.69*** (1.59–1.80)	1.61*** (1.52–1.72)
Four or more births			2.25*** (2.11–2.39)	2.04*** (1.91–2.17)
Mass media				
No			Ref	Ref
Yes			0.65*** (0.63–0.67)	0.73*** (0.71–0.76)
Health insurance coverage				
No			Ref	Ref
Yes			0.44*** (0.41–0.47)	0.46*** (0.43–0.49)
Religion				
Christians			Ref	Ref
Islam			1.61*** (1.55–1.67)	1.82*** (1.74–1.89)
Traditional			1.39*** (1.28–1.52)	1.72*** (1.57–1.88)
No/other			2.46*** (2.29–2.64)	2.41*** (2.24–2.56)
ANC visits				
Less than 4			Ref	Ref
4 or more			0.32*** (0.31–0.33)	0.33*** (0.32–0.34)
Sex of household head				
Male			Ref	Ref
Female			0.84*** (0.81–0.88)	0.83*** (0.79–0.87)
Healthcare decision-making capacity				
Alone			Ref	Ref
Not alone			0.96* (0.92-1.00)	0.95** (0.91–0.99)
Wealth index				
Poorest			Ref	Ref
Poorer			0.76*** (0.74–0.79)	0.81*** (0.79–0.84)
Middle			0.62*** (0.60–0.65)	0.69*** (0.67–0.72)
Richer			0.52*** (0.49–0.55)	0.61*** (0.58–0.64)
Richest			0.32*** (0.29–0.36)	0.44*** (0.40–0.49)
*Community-level variables*				
Accessing healthcare				
With big problem				Ref
Without a big problem				0.84*** (0.81–0.88)
Community literacy level				
Low				Ref
Medium				0.75*** (0.72–0.88)
High				0.69*** (0.66–0.71)
Community socioeconomic status				
Low				Ref
Moderate				0.43*** (0.40–0.46)
High				0.79*** (0.75–0.82)
Sub-region				
West Africa				Ref
Central Africa				2.09*** (1.98–2.19)
East Africa				1.55*** (1.48–1.62)
Southern Africa				1.31* (1.19–1.45)
Weighted sample (N)		**103**,**011**	**103**,**011**	**103**,**011**
Pseudo R^2^		0.0055	0.1618	0.1794

^*^p < 0.05, ^**^p < 0.01, ^***^p < 0.001, Ref-Reference category

Sampling weights (v005/1,000,000) were applied to all analyses to account for the DHS complex survey design, including stratification, clustering, and unequal probabilities of selection. For the pooled analysis across the 28 countries, datasets were appended and country-specific sampling weights were applied without cross-country normalization, consistent with standard DHS practice. Survey design variables including primary sampling units (PSUs; v021) and strata (v022/v023) were retained, with strata and clusters made unique across countries to ensure correct variance estimation. This approach is consistent with standard DHS pooled analysis methodology [26, 44], where weights are used to ensure each country’s sample remains nationally representative of its own population, and the pooled sample reflects the actual survey sample sizes rather than the relative population sizes of the countries. Multicollinearity was checked using the variance inflation factor (VIF). We had a mean VIF of 1.31, ranging from 1.03 to 2.00. The results showed no evidence of multicollinearity.

## Results

### Prevalence of home delivery

The prevalence of home delivery among women in rural areas of Africa was 34.01% [95% CI: 23.33%-35.26%]. The country-specific prevalence indicated that Chad had the highest (85.19%) prevalence, while Rwanda had the lowest (5.91%) prevalence (see Fig. 2).

### Cross-tabulation results between explanatory variables and home delivery among rural women in Africa

In Table 2, this study revealed that the highest prevalence of home delivery occurred among rural women who had planned pregnancies (35.8%), were aged 45–49 (40.7%), had no formal education (46.4%), had husbands with no formal education (45.4%), were married (35.6%), were not working (41.7%), had four or more births (38.9%), were not exposed to mass media (43.0%), and had less than four ANC visits (48.4%). The highest proportion of home delivery was found among women who had not subscribed to health insurance (36.1%), those who did not make decisions alone (34.8%), those with no/other religion (54.9%), those with big problems accessing health facilities (48.8%), those living in low community literacy levels (45.5%), and those with low community socioeconomic status (39.8%). Regarding wealth status and sex of household heads, the highest proportion was recorded among women in the poorest wealth quintile (44.3%) and male household heads (35.1%), respectively. Regarding the subregion, women in Central Africa had the highest proportion of home deliveries (44.5%), whereas those in Southern Africa had the lowest (21.8%). All variables were identified as statistically significant.

### Association between pregnancy intention and home delivery among women in rural Africa adjusting for risk factors

Table 3 presents the results of the predictors of home delivery among women in areas of Africa. In the final model, the study revealed that, compared to women who planned pregnancy, those with mistimed pregnancy [aOR = 0.79, 95% CI = 0.76–0.82] and those with unwanted pregnancy [aOR = 0.86, 95% CI = 0.81–0.92] had lower odds of home delivery. Women aged 35–39 [aOR = 0.61, 95% CI = 0.56–0.67], those who had secondary/higher education [aOR = 0.71, 95% CI = 0.68–0.74], those whose partners had secondary/higher education [aOR = 0.80, 95% CI = 0.76–0.84], and cohabiting women [aOR = 0.82, 95% CI = 0.79–0.86] had lower odds of having home delivery compared to those aged 15–19, those with no formal education, those whose partners had no formal education, and married women, respectively. Regarding employment status and mass media, women who were working [aOR = 0.80, 95% CI = 0.78–0.83] and those who were exposed to mass media were less likely to have home delivery [aOR = 0.73, 95% CI = 0.71–0.76] compared to those who were not working and not exposed to mass media, respectively.

It was found that women who have subscribed to health insurance were less likely to have home delivery [aOR = 0.46, 95% CI = 0.43–0.49] compared to those who have not subscribed to health insurance. Compared to women with less than 4 ANC visits and male household heads, those who had four or more ANC visits [aOR = 0.33, 95% CI = 0.32–0.34] and female household heads [aOR = 0.83, 95% CI = 0.79–0.87] had lower odds of home delivery. Women who do not make healthcare decisions alone were found to have a lower likelihood of home delivery [aOR = 0.95, 95% CI = 0.91–0.99] compared to those who make healthcare decisions alone.

Regarding wealth status, the odds of home delivery decrease with an increased wealth quintile. Particularly, women in the richest wealth quintile [aOR = 0.44, 95% CI = 0.40–0.49] had a lower likelihood of home delivery compared to women in the poorest wealth quintile.

Women without a big problem of accessing a health facility [aOR = 0.84, 95% CI = 0.81–0.88]; those who lived in communities with high literacy levels [aOR = 0.69, 95% CI = 0.66–0.71]; and those who lived in communities with moderate socioeconomic status [aOR = 0.43, 95% CI = 0.40–0.46] had the lowest odds of home delivery compared to those with a big problem of accessing a health facility, low community literacy level, and low community socioeconomic status, respectively. The odds of home delivery increase with increasing parity. Specifically, women with four or more births [aOR = 2.04, 95% CI = 1.91–2.17] had a greater likelihood of home delivery compared to those with one birth. The likelihood of home delivery was higher among those with no/other religion [aOR = 2.41, 95% CI = 2.24–2.56] and those in Central Africa [aOR = 2.09, 95% CI = 1.98–2.19] compared to Christians and those in West Africa, respectively.

### Pseudo R^2^

The three distinct pseudo-R² values from the logistic regression models offer valuable insights into how effectively each model accounts for variations in the dependent variable. In Model I, which includes only the key independent variable, the lower pseudo-R² indicates that this single factor accounts for just a small part of the outcome variability. Model II, which incorporates both the key independent and individual-level variables, exhibits a significant increase in the pseudo-R² (0.1618), suggesting that these added factors substantially improve the model’s explanatory capability. Model III, which encompasses all variables, reaches the highest pseudo-R² (0.1794), indicating that this complete set of predictors yields the best fit. The rising pseudo-R² values across the models imply that each additional set of variables aids in explaining the outcome, with the complete model showing the strongest performance.

## Discussion

This study examined the prevalence and determinants of home delivery among rural women across 28 African countries. Home delivery which remains a critical indicator of maternal and child health, is often observed alongside limited access to skilled birth attendance and an increased risk of adverse maternal and neonatal outcomes [7, 45–47]. Understanding the factors associated with home delivery is therefore essential for informing targeted interventions aimed at improving maternal healthcare utilization.

Our analysis showed a 34.01% prevalence of home delivery among women in rural areas of Africa, meaning that more than one in four births occurred at home. This prevalence is higher than previously reported estimates in East Africa (23.68%) [48], Africa (23.67%) [49], and India (22%) [50], although lower than the 88.3% reported in Ethiopia [24]. The high prevalence of home delivery among rural women in Africa may be related to various structural and systemic issues, such as the inability of many African countries to fulfill the Abuja Declaration goal of allocating at least 15% of their gross domestic product (GDP) to healthcare [51], a shortage of healthcare personnel due to medical brain drain, insufficient healthcare infrastructure, an unequal emphasis on deliveries at urban health facilities, and persistent political unrest [51, 52].

Substantial disparities were observed across countries, with prevalence ranging from 5.91% in Rwanda to 85.19% in Chad. The high prevalence in Chad is consistent with previous findings [53] and this is comparable to other studies conducted in low- and middle-income countries [9, 54]. This may reflect lower educational attainment, reduced wealth, limited access to media, and higher parity among women [53, 55]. In contrast, the low prevalence observed in Rwanda may coincide with extensive reforms in its health system aimed at decreasing the number of home births and enhancing maternal health outcomes. From 2000 to 2010, Rwanda established policies promoting facility-based childbirth, implemented performance-based financing, and introduced community health insurance [56]. These findings underscore the importance of context-specific strategies in addressing home delivery across different settings [57].

Factors associated with higher odds of home delivery (aOR > 1, *p* < 0.05) included higher parity, affiliation with no/other religion, and residence in Central Africa. Conversely, factors associated with lower odds of home delivery (aOR < 1, *p* < 0.05) included mistimed or unwanted pregnancy intention, older maternal age, higher educational attainment, exposure to mass media, health insurance coverage, increased antenatal care visits, higher household wealth, and improved access to healthcare.

The study’s binary logistic regression analysis revealed that parity, religion, and sub-region are significant risk factors associated with home delivery among women in rural Africa. However, pregnancy intention, age, educational level, partner’s educational level, marital status, employment status, exposure to mass media, health insurance coverage, number of ANC visits, sex of household head, health decision-making capacity of the women, wealth index, community literacy level, and community socio-economic status were identified as significantly protective factors against home delivery among women in rural Africa.

### Protective factors associated with lower odds of home delivery

The findings revealed that pregnancy intention is associated with women’s choice of place of delivery in Africa. Specifically, this study indicates that rural women who had mistimed pregnancy intentions had lower odds of home delivery. Interestingly, our results, in contrast with some previous findings [5, 18, 19, 58, 59], showed that women who had mistimed pregnancy intention were less likely to report home delivery than women with planned pregnancy intention.

Several factors may help explain why women with mistimed pregnancies opt for home delivery. One possible explanation could be due to the confidence associated with psychological readiness for pregnancy, potentially reducing or eliminating the anxiety that often accompanies unintended pregnancies [22, 60]. Such anxiety could be associated with lower home delivery due to fear [60, 61].

In this study, the odds of home delivery were lower among older women aged 20–49. This is supported by previous studies [48, 62], which indicate that older women are less likely to opt for home delivery. Women of these age groups may be more likely to decide for themselves because they are often financially independent or married, which may also influence their delivery place preferences. To increase understanding of these issues, further research is recommended.

Our results, supporting previous findings [8, 9, 16, 63], indicate that educational level and community literacy level are significantly associated with home delivery. Specifically, women and their partners with secondary education or higher are less likely to choose home delivery. This finding underscores the strong association between education and economic and health advancement [64]. Education is consistently associated with better health and economic outcomes in every society; therefore, prioritizing education, particularly for the girl child, rather than arranging early marriages, may represent a vital strategy for improving the healthcare and well-being of mothers and children [65]. This is because education for the girl child can effectively combat early marriage and its negative consequences [66]. Furthermore, the WHO has recommended a systematic and multisectoral approach to address the disproportionate maternal deaths occurring in the African region [7].

Rural women in Africa who are exposed to mass media are less likely to choose home delivery compared to those who are not. Other studies conducted in Africa [24, 48, 67] support this finding. One potential explanation for the lower likelihood of home deliveries among rural women in Africa is that these women receive information from mass media channels such as radio, television, and newspapers, which generally raise awareness and knowledge about pregnancy, promote hospital deliveries, and provide insights on complications associated with childbirth at home [49]. Furthermore, media exposure may necessitate changes in women’s attitudes, social norms, and behaviours that facilitate access to healthcare facilities, resulting in a decreased likelihood of home deliveries [68]. The impact of mass media on rural women’s decisions regarding where to give birth underscores the need for enhanced access to mass media resources.

Further, health insurance subscription was found to be an important driver of home delivery among rural women in Africa. The odds of home delivery were lower among rural women in Africa who had subscribed to health insurance. This is supported by a finding in Africa [48]. This may be attributed to increased awareness and knowledge regarding the benefits of health insurance [9]. Another possible reason could be that women who have health insurance tend to experience fewer delays in seeking, travelling to, and receiving care at a hospital compared to those who pay out of pocket [69]. Hence, rural women with health insurance coverage are also more inclined to choose to give birth in health facilities [70].

The current study revealed that ANC visits are an important predictor of home delivery. Our study indicates that rural women in Africa who had four or more ANC visits had a lower likelihood of home delivery. This observation aligns with findings from research carried out in Africa [48], Ethiopia [22, 24, 71, 72], Ghana [73], and Madagascar [74]. The ANC package encompasses health education and initiatives concerning pregnancy, complications, and recommended practices [75]. Given that the study was conducted among rural women in Africa, access to information on maternal and related health topics for the community, including mothers, is primarily limited to health facilities [49]. Therefore, the local health office needs to enhance the utilization of ANC services.

Female household heads had lower odds of home delivery, which is consistent with findings in previous studies conducted in Africa [9, 55, 76]. This result may be linked to the observation that when women have control, they often make choices that prioritize their understanding of maternal health and appreciate the importance of effectively using maternal healthcare to safeguard their lives and the lives of their infants [77]. Also, our study revealed that rural women who did not make healthcare decisions alone were less likely to give birth at home. This finding is reinforced by other empirical evidence in Africa [49, 78, 79]. The choice of where to give birth necessitates decision-making by mothers and their immediate family members [49]. Given that women often serve as the primary caregivers, empowering them to make significant health-related decisions can lead to improved outcomes.

The study revealed that wealth status was an important factor associated with home delivery. In our current study, rural women in the richest wealth status quintile were less likely to give birth at home, which aligns with findings from other research studies conducted in Africa [49, 59, 80–82] and Nepal [83]. The plausible reason could be that richer women are more likely to afford transportation costs and the need to purchase additional supplies for giving birth in a healthcare setting [49, 84]. It is essential to consider implementing free transportation services using ambulances to ensure better access to healthcare facilities. In addition, at least rural women in medium socioeconomic status had lower odds of home delivery. This is consistent with studies conducted in Africa [37, 74, 85] and Bangladesh [86].

Another significant factor found to be statistically associated with home delivery among rural women in Africa was the distance to a health facility. The findings of our study, as well as those of previous studies, also have implications for improving access to and utilization of health facility delivery in rural Africa. In line with previous evidence in Africa [23, 24, 84, 87], we found that women who experience difficulties accessing health facilities are more likely to report home delivery. Therefore, addressing the barriers posed by availability, accessibility, and affordability may improve facility-based delivery, thereby reducing the likelihood of home delivery and the potential adverse outcomes associated with it. The issue of availability, accessibility, and affordability is central within the rural context, as there is always an existing disparity in healthcare access among urban and rural residents [11–14]. Addressing this disparity would also involve making maternal healthcare services equitably available, accessible, and affordable for rural dwellers.

### Risk factors associated with higher odds of home delivery

Another crucial factor influencing home delivery identified in this study was parity. It was found that the odds of home delivery increase with increasing parity. This finding is congruent with other studies in Eastern Africa [24, 48]. Women in rural areas of Africa often view childbirth as a normal event; they may be less inclined to seek medical assistance during labour [48, 88]. Additionally, the existing literature suggests that past experiences with healthcare facilities play a role in this decision; negative experiences from previous deliveries can deter women from accessing care for future births [89, 90]. Thus, having less anxiety about complications and adverse care experiences during childbirth may lead women to avoid using health services in subsequent pregnancies [48].

An important and seemingly paradoxical finding from our analysis is that while higher parity was associated with increased odds of home delivery, older maternal age was associated with decreased odds. These two factors are often correlated, as multiparous women tend to be older; however, the opposing directions of association suggest that parity and age capture distinct dimensions of childbirth behavior. One possible explanation is that parity primarily reflects accumulated birth experience, which may contribute to women perceiving childbirth as a routine, low-risk event, thereby reducing the perceived need for facility-based delivery [48, 88]. In contrast, older women may have had more opportunities to experience or witness birth complications, which may heighten risk perception and preference for facility-based delivery.

The religious affiliation of women has been observed to significantly influence home delivery, with rural women in Africa identifying with other religions or having no religious affiliation being more inclined to have home deliveries compared to women who are Christians. This aligns with previous studies in Africa [90, 91]. This indicates that religion is associated with home delivery. It is important to recognize that being affiliated with a religion entails beliefs and practices that may impact women’s overall maternal health behaviours [90, 92].

The reported results indicated that the marital status of rural women in Africa was significantly associated with home delivery. It was found that cohabiting women in rural areas of Africa were less likely to have home delivery compared to married rural women. This is inconsistent with a similar study in Benin [93]. A plausible reason could be that women who become pregnant outside of marriage may be less inclined to seek out formal healthcare services due to societal stigma and exclusion, as well as the biased attitudes of healthcare professionals [94]. Employment status has been reported as a significant determinant of home delivery by past studies in Bangladesh [95]. In our analysis, it was discovered that rural women who were working were less likely to have home delivery. This is inconsistent with results that have been obtained in Pakistan [94] and Bangladesh [95].

Finally, it was found that the sub-region showed a significant statistical association of home delivery rates among rural women in Africa. Rural women living in Central and Eastern Africa were 2.09 times and 1.55 times more likely to have home deliveries compared to their counterparts in West Africa, respectively. This corresponds with findings from previous research conducted in Africa [87]. This trend may reflect prevailing social customs in Central and Eastern Africa [48]. Additionally, accessibility and quality of maternal health care services, along with health policies in West Africa, could contribute to the reduction in home deliveries [96].

### Strengths and limitations of the study

The study has several strengths and some limitations. One of the key strengths of this study is the wide-ranging scope, covering 28 countries across Africa. This broad geographical coverage allows for a more representative understanding of the relationship between pregnancy intention and home delivery practices among rural women in the region. By including diverse countries, the study captures variations in socio-economic and healthcare contexts, contributing to the generalizability of the findings. Furthermore, the study used high-quality data sourced from nationally representative Demographic and Health Surveys (DHS). The application of advanced statistical analytical techniques further strengthens the credibility and reliability of the findings and provides a solid foundation for future comparative research.

However, there are some limitations to consider. First, reliance on secondary data sources may introduce inconsistencies or gaps in data availability and quality across countries. Additionally, due to the cross-sectional nature of the DHS data, causal inferences between pregnancy intention and home delivery cannot be established; the findings should therefore be interpreted as associations rather than causal relationships.

Another important limitation is the temporal spread of the data (2011–2024). While this provides a large pooled sample, it assumes that the relationships between pregnancy intention, covariates, and home delivery have remained stable over time. This assumption may not hold, given substantial changes in maternal health policies, healthcare infrastructure, and communication technologies across many African countries during this period. Consequently, findings from countries with older surveys (e.g., Congo 2011–12, Comoros 2012, Gabon 2012) may not reflect more recent developments. The estimated odds ratios should therefore be interpreted as average effects across this timeframe, and the temporal spread may mask evolving trends and country-specific progress.

Importantly, this study revealed substantial heterogeneity in home delivery prevalence across the 28 countries, ranging from 5.91% in Rwanda to 85.19% in Chad. This wide variation reflects underlying differences in health systems, policy environments, cultural norms, and socio-economic conditions across settings. While the pooled analysis provides an overall regional estimate for rural Africa, these aggregated results represent average patterns and may obscure meaningful country-specific risk profiles and contextual nuances. As such, the pooled estimates and odds ratios should not be interpreted as uniformly applicable across all countries. Future research would benefit from country-specific or sub-regional analyses, as well as multilevel modeling approaches that explicitly account for the hierarchical structure of the data and allow for the examination of both within- and between-country variation.

Additionally, the study lacks direct measurement of cultural norms and beliefs surrounding childbirth practices, as such variables are not captured in DHS datasets. Although some community-level indicators offer indirect insights, they do not fully reflect the influence of cultural factors on delivery decisions. Future studies could employ qualitative or mixed-methods approaches to better understand these dynamics.

Finally, the study focuses specifically on rural women in Africa, which may limit the applicability of the findings to urban populations or other global contexts. To enhance external validity, future research could examine similar relationships in different settings and populations. Further work could also explore DHS data by specific survey waves to enable more robust trend analyses over time.

## Conclusion and recommendations

This study shows that home delivery remains prevalent among rural women in Africa (34.01%), with substantial between-country disparities ranging from 5.91% in Rwanda to 85.19% in Chad. Key risk factors include high parity, affiliation with no or other religion, and residence in Central Africa, while antenatal care utilization, higher education, and health insurance coverage were protective.

These findings highlight the need for context-specific interventions. In high-prevalence settings, particularly in Central Africa and countries such as Chad, efforts should prioritize structural and system-level interventions, including expanding access to skilled birth attendance, strengthening rural health infrastructure, improving transportation systems, and targeting multiparous and underserved populations. In contrast, in lower-prevalence settings, such as Rwanda, interventions should focus on sustaining gains by addressing residual inequities through improved health insurance coverage, enhanced antenatal care utilization, and strengthened health education.

Across all contexts, expanding maternal health services in underserved communities, addressing religious and sociocultural barriers, and investing in women’s education and economic empowerment remain essential for reducing home delivery and improving maternal health outcomes.

## Data Availability

The dataset used is freely available at https://dhsprogram.com/data/available-datasets.cfm.

## Abbreviations

aOR: Adjusted odds ratio
CI: Confidence intervals
DHS: Demographic and health Survey
LMICs: Low-and-middle-income countries
MMR: Maternal Mortality Ratio
Ref: Reference category
VIF: Variance Inflation Factor
WHO: World Health Organization
